# When phage restriction is not immunity: Immune gates, replication-niche barriers, and assay-limited phenotypes

**DOI:** 10.1016/j.engmic.2026.100295

**Published:** 2026-07-30

**Authors:** Yong Everett Zhang

**Affiliations:** Department of Biology, University of Copenhagen, DK-2200, Copenhagen, Denmark

**Keywords:** Immune gating, Phage adsorption, Replication-niche barriers, Phage-restrictive phenotypes, Stress-program barriers, Microbial engineering

## Abstract

Antiphage discovery increasingly yields restrictive phenotypes before mechanisms are known. This Perspective argues that reduced plaque formation or low efficiency of plating should be classified phenotype-first, not automatically as immunity. I distinguish immune-gated defenses, replication-niche barriers, and assay-limited phenotypes, and propose a decision-tree workflow using native context, matched physiology, infection-stage readouts, triggers, and phage-mediated rescue. Clearer labels should broadly improve annotation, counter-defense interpretation, and phage-host engineering for therapy, biocontrol, and biotechnology in complex microbial settings.

## Graphical abstract

**Figure:**
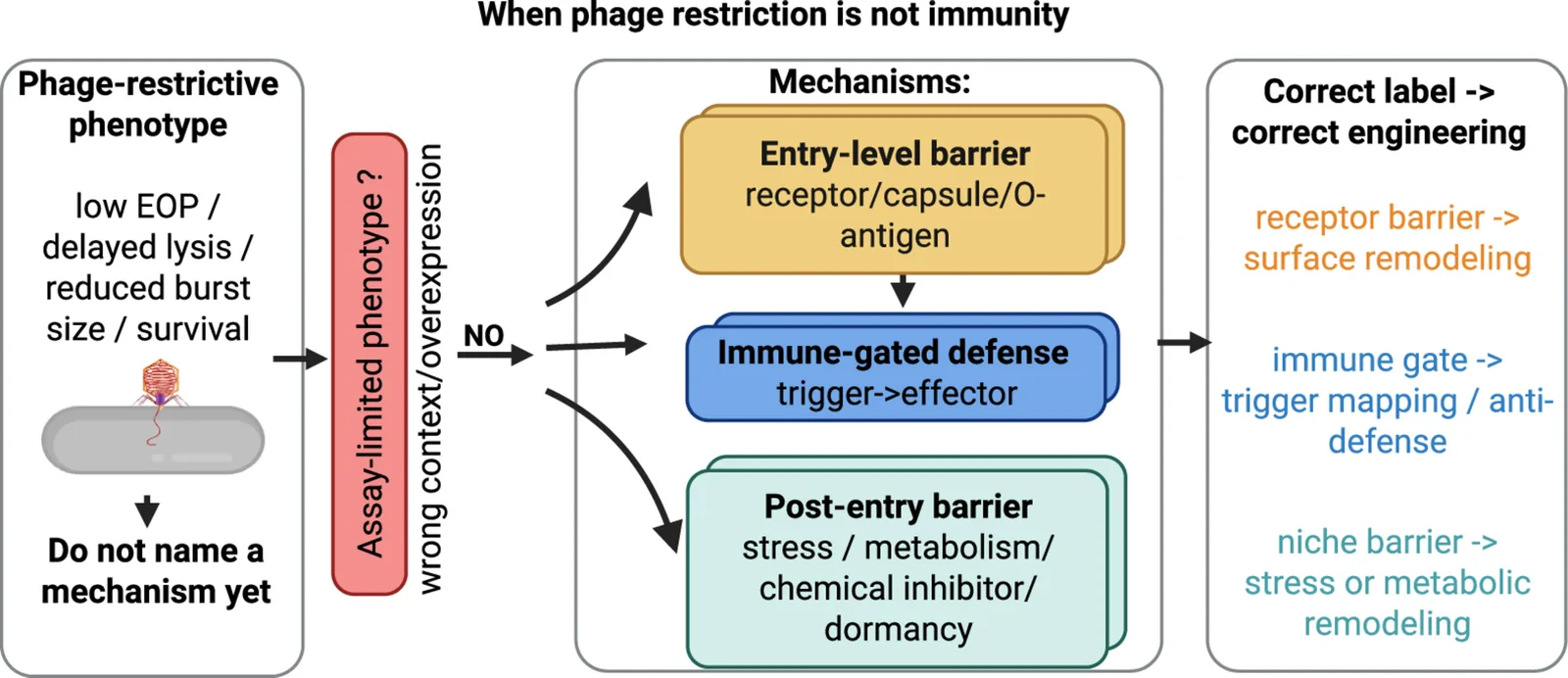

## 1 Importance

High-throughput screens often measure phenotype before mechanism. A lower efficiency of plating, delayed lysis, or improved host survival can reflect an immune-gated defense, a replication-niche barrier, or a phenotype shaped mainly by the assay itself. Replication-niche barriers deserve special emphasis because they operate at the level of infection permissivity: receptor and surface states determine whether adsorption and genome delivery can occur, while post-entry physiological states determine whether a cell can become a productive viral factory. If entry fails, intracellular immune defenses are not engaged in that infection event; if entry succeeds, the phage must still evade immune gates and remodel stress- or metabolism-based barriers. From an engineering perspective, these distinctions change the interventions (e.g., receptor remodeling, niche construction, or immune evasion) and should make phage-defense annotation, counter-defense biology, and engineering-oriented validation more reproducible across studies.

Bacterial antiphage research has entered a discovery-scale era. Defense-island mining, functional screening, heterologous cloning, and machine-learning prediction have successfully moved the field beyond a small set of canonical systems, such as restriction-modification and CRISPR-Cas, into a rapidly expanding catalog of candidate and validated antiphage loci [[1], [2], [3], [4], [5], [6]]. Precisely because these approaches now generate candidates faster than mechanisms can be resolved, phenotype-first reporting and staged mechanistic validation are becoming increasingly important. In practice, reduced plaque formation, lower efficiency of plating (EOP), delayed lysis, or improved host survival is still often narrated through a single word, *defense*, even though these readouts do not by themselves reveal the underlying mechanism.

The key point is that phage restriction is a phenotype, not yet a mechanism. A restrictive phenotype can arise because a bacterium encodes a dedicated immune system that recognizes infection or non-self. It can also arise because the host cell is a poor viral replication niche: the receptor is masked by surface polysaccharides, the chemical environment contains inhibitory small molecules, biosynthetic capacity is limiting, or stress physiology has shifted the cell into a survival-oriented state. A third possibility is experimental: the phenotype may be heavily conditioned by the assay itself, for example through non-native expression level, non-native timing, altered stoichiometry, or generic growth burden. Treating all three cases as "defense" makes discovery look cleaner than it really is, but it blurs mechanism and hampers the rational engineering of phage-host interactions.

I therefore suggest a simple vocabulary for separating these outcomes. **Immune-gated defenses** are dedicated antiphage systems in which a recognition step decides when restriction should occur. The recognition step - the gate - can be a self/non-self discrimination system (as in restriction-modification, where methylation marks self-DNA) or an infection-triggered activation switch (as in systems that sense a phage protein before unleashing a toxic effector). **Replication-niche barriers** are host or environmental states that make a living bacterial cell non-permissive, or only weakly permissive, for adsorption or phage production without yet demonstrating a dedicated immune gate. They can be subdivided into entry-level barriers (which prevent phage access to the cytoplasm, such as surface polysaccharides that block adsorption) and post-entry barriers (which restrict phage multiplication inside the cell, including limitations of biosynthetic machinery and stress-response programs). **Assay-limited phenotypes** are apparent protective phenotypes that change meaning, weaken, or disappear when native host context, timing, expression level, or physiology is restored. This vocabulary is not meant to downgrade non-immune forms of restriction. Rather, it keeps biologically real barriers distinct from dedicated immunity and from assay-generated artifacts, and it encourages engineers to ask what, exactly, a phage must overcome to replicate in a given host background.

**Fig. 1:**
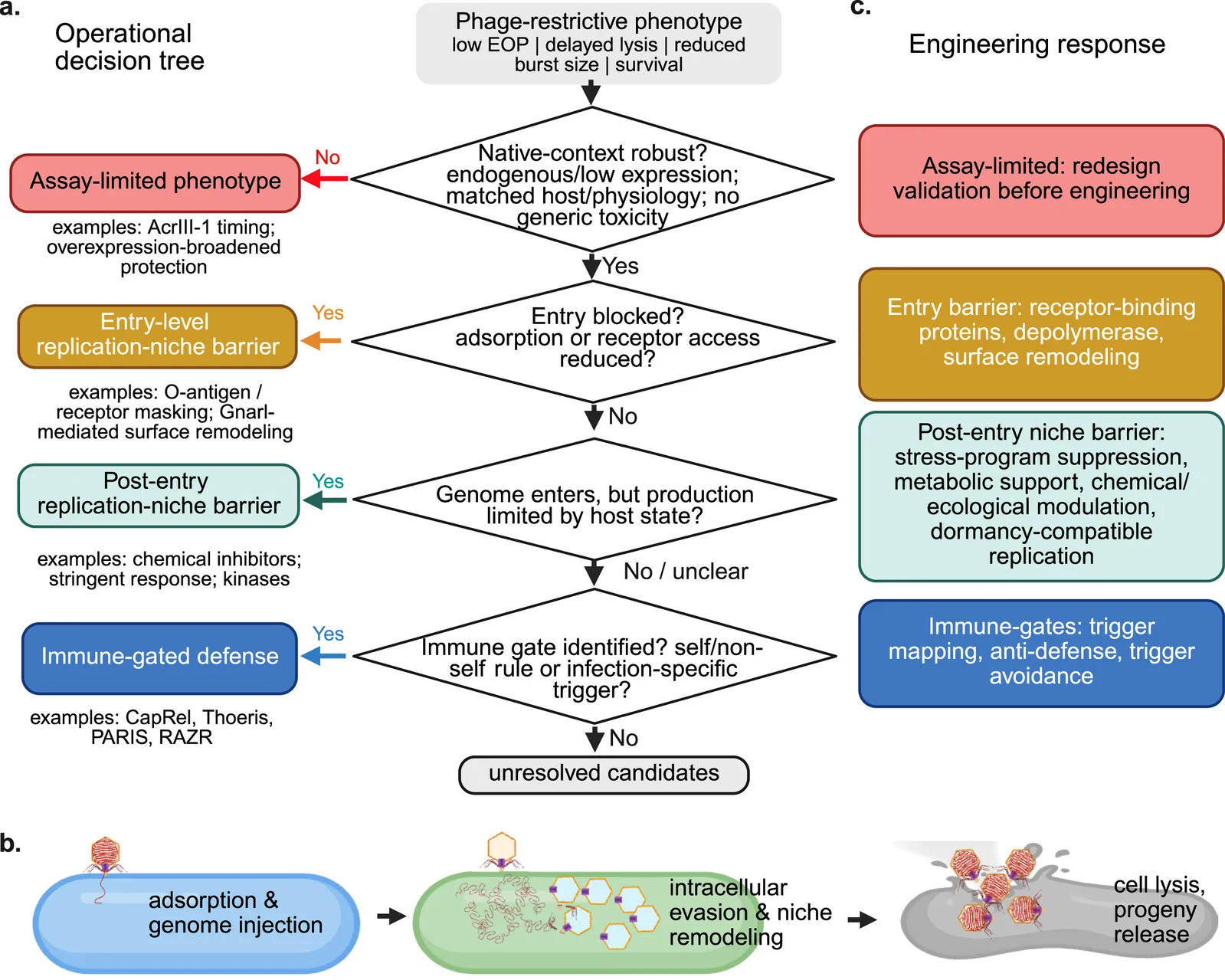
**A stage-based decision workflow for classifying phage-restrictive phenotypes**. (**a**) Operational decision tree for assigning mechanistic labels to an observed phage-restrictive phenotype, such as low efficiency of plating, delayed lysis, reduced burst size, or improved host survival. The first question is whether the phenotype is robust in a native or near-native context, with endogenous or low expression, a matched host background or physiology, and no generic toxicity. Phenotypes that fail this test are best treated as assay-limited. Native-context phenotypes are then separated by infection stage. Reduced adsorption or receptor access indicates an entry-level replication-niche barrier. Successful genome entry followed by impaired phage production indicates a post-entry replication-niche barrier when the block is linked to host physiology, chemical or ecological inhibition, stress signaling, dormancy, or metabolic limitation. The label immune-gated defense should be reserved for cases in which restriction is licensed by self/non-self discrimination or an infection-specific trigger. Phenotypes that do not yet meet these criteria should remain unresolved candidates. (**b**) Phage restriction can be mapped onto successive stages of infection. Receptor and surface states determine whether adsorption and genome delivery occur; intracellular immune gates and replication-niche barriers become relevant only after entry; and productive infection ultimately requires intracellular remodeling sufficient for virion production and progeny release. This stage-based view emphasizes that receptor or entry barriers act upstream of intracellular immunity, whereas post-entry physiological barriers determine whether a cell becomes a productive viral factory. (**c**) The engineering response depends on the mechanism. Assay-limited phenotypes require redesigned validation before engineering conclusions are drawn. Entry barriers point to receptor-binding proteins, depolymerases, or surface remodeling. Post-entry replication-niche barriers call for niche remodeling, such as stress-program suppression, metabolic support, chemical or ecological modulation, or dormancy-compatible replication. Immune-gated defenses instead require trigger mapping, anti-defense strategies, or trigger avoidance. Representative examples are discussed in the text. (Created in BioRender. ZHANG, Y. (2026) https://BioRender.com/rud90dy).

These categories should be read as operational labels for the dominant demonstrated layer, not as mutually exclusive compartments. A single infection can encounter surface barriers, physiological barriers, and immune gates in series, and a single locus may couple immune recognition to host physiology. In such cases, the label should identify the mechanism best supported by the evidence: whether restriction is licensed by a recognition rule, whether permissivity fails because of surface or physiological state, or whether the phenotype depends mainly on assay context. Thus, a system activated by infection-associated resource depletion can be described as a stress-coupled immune gate when a dedicated antiphage locus senses that cue and activates an effector, as illustrated by nucleotide-coupled Gabija activation [7], whereas the same metabolic condition without a dedicated sensor-effector module is better described as a replication-niche barrier. Fig. 1**a** converts this vocabulary into an operational decision tree. Starting from a phage-restrictive phenotype, the workflow first asks whether the phenotype survives native-context and matched-physiology controls, then separates entry-level receptor barriers from post-entry niche restrictions, and finally reserves the label immune-gated defense for cases in which restriction is licensed by a self/non-self rule or an infection-specific trigger.

This stage-based view is especially important because receptor barriers act upstream of intracellular immunity: if adsorption or genome delivery fails, cytoplasmic immune gates may never be engaged in that infection event. If entry succeeds, the phage must still evade immune gates and overcome post-entry physiological barriers before productive replication can occur (Fig. 1**b**). The value of this vocabulary is that it raises the evidentiary standard for immunity without dismissing other forms of phage restriction. Among the three categories, immune-gated defense is the strongest and narrowest mechanistic claim. It should therefore be tested by the clearest mechanistic criteria, rather than inferred from plaque reduction alone. The point is not to make immunity an unattainable label, but to reserve it for cases in which restriction is coupled to a recognizable gate: a self/non-self distinction or an infection-triggered activation step.

## 2 Immune-gated defenses require recognition

Immune-gated defenses are dedicated antiphage systems in which recognition controls restriction. The gate can be constitutive self/non-self discrimination, as in restriction-modification systems and CRISPR-Cas, where methylation state or guide-RNA complementarity helps distinguish host from invader [2,4]. It can also be infection-triggered activation, as in many recently discovered systems whose effectors may be broad, toxic, or suicidal but are kept inactive until a phage-derived cue is detected [[8], [9], [10], [11], [12], [13]]. In this sense, the immune label depends less on whether the effector is gentle or chemically specific, and more on whether destructive activity is conditionally licensed by recognition.

This definition also helps handle boundary cases without forcing them into rigid boxes. Constitutively expressed toxin-antitoxin or abortive-infection modules should not be classified by expression mode alone. They qualify as immune-gated defenses when toxin activation is conditionally licensed by an infection-associated event, such as a phage protein, phage-mediated host shutoff, selective antitoxin destabilization, or another defined infection-process cue. If the same module is activated only by generic starvation or non-infection stress, the more conservative label is a stress-program replication-niche barrier or an unresolved replication-niche effect [3]. Gabija-like nucleotide-sensing systems occupy a similar interface [7]. When a dedicated GajA-GajB module senses infection-associated nucleotide or ATP depletion and activates nuclease-mediated abortive infection, it is best described as a stress-coupled immune gate; when low nucleotide availability simply reflects poor host physiology without a dedicated sensor-effector module, it is better described as a growth-machinery limitation within the replication-niche barrier category.

Direct phage-cue sensing provides the clearest version of immune gating. The CapRel system in *E. coli* contains a sensor-effector architecture: a newly synthesized phage major capsid protein binds the CapRel sensor, relieves autoinhibition, and activates a toxSAS enzyme that pyrophosphorylates tRNAs, arrests translation, and aborts productive infection [9]. A later study showed that the same sensor can recognize a second, unrelated phage protein through an overlapping but distinct interface, demonstrating that one immune gate can integrate multiple viral cues [11]. The Sau-Thoeris system in staphylococci operates on a similar principle: phage major head proteins bind ThsB sensors, which initiate cyclic signaling and NAD⁺ depletion through the Thoeris pathway, killing the infected cell before the phage can complete its cycle [12].

Other systems illustrate different versions of the same principle. PARIS functions as a reserve defense activated not by a structural component of the incoming phage but by a phage counter-defense protein: the anti-restriction protein Ocr binds the AriA antitoxin and releases the AriB toxin, converting phage anti-defense activity into an immune trigger [10]. RAZR adds a supramolecular form of gating: a recombination protein in one phage and portal proteins in others form ring-like scaffolds with similar geometry, promoting oligomerization of the RAZR sensor and activation of its RNase effector [13]. More broadly, a phage-protein screen that expressed individual phage open reading frames in diverse *E. coli* backgrounds identified many candidate trigger-defense pairs, supporting a sensor-trigger-effector view of bacterial immunity [8].

These examples clarify a common confusion. An immune effector does not need to attack a phage-specific molecule. Many genuine systems terminate infection by depleting NAD⁺, damaging RNA, stopping translation, or arresting growth. What makes them immune is the gate that keeps the effector off before infection and turns it on after the appropriate phage-derived cue. This is why NAD-depleting defenses should not be dismissed as generic toxicity: phages have evolved dedicated pathways to rebuild NAD⁺ from defense-generated degradation products, showing that these systems impose recurrent and mechanistically meaningful selective pressure on viruses [14]. Broad biochemical execution is compatible with genuine immunity when it is coupled to immune gating.

## 3 Replication-niche barriers are real biology, not failed immunity

If dedicated immunity is defined by immune gating, an important middle category comes into focus: biologically relevant host states that restrict phage propagation even when no dedicated immune sensor has been shown. These states are referred to here as replication-niche barriers because they make a living bacterial cell non-permissive, or only weakly permissive, for viral entry, genome replication, protein synthesis, assembly, or release. This category captures the fact that phages must construct a replication-permissive niche, not merely evade immune sensors. In many natural and applied settings, replication-niche barriers may be at least as important as immune-gated defenses, and sometimes more immediately determinant, because they control whether infection begins and whether virion production can be sustained.

**Entry-level barriers: the cell surface as the first gatekeeper.** Bacterial physiology shapes receptor structure, abundance, modification, and accessibility through envelope composition, O-antigen or capsule state, biofilm matrix, nutrient status, and stress-dependent surface remodeling. These properties determine whether a phage can adsorb and inject its genome, and therefore whether intracellular immune systems and post-entry barriers become relevant in that infection event. In wild *E. coli* isolates, O-antigen and related lipopolysaccharide features can obstruct receptor access and limit adsorption by multiple phages. Recent work illustrates both the strength and the complexity of this surface layer: several phage accessory genes, including *gnarl1–3*, alter O-antigen structures, and Gnarl3 associates with the UDP-glucose biosynthesis enzyme GalU, producing envelope changes that can potentiate infection by phages such as T5 and, in some strain contexts, T4[15]. Once O-antigen barriers are genetically removed or experimentally bypassed, intracellular restriction systems that were previously hidden can become visible. The surface layer therefore determines whether a phage can reach the inside of the cell at all, but it is not automatically a dedicated immune sensor. It is better described as an entry-level replication-niche barrier that can complement, obscure, or precede immune-gated defenses.

Phase-variable surface structures extend this idea to dynamic and reversible entry barriers. In *Bacteroides thetaiotaomicron*, phase-variable capsular polysaccharides, lipoproteins, and related surface structures modify phage susceptibility, allowing non-permissive surface states to be selected under phage predation while reversible population heterogeneity can maintain susceptible variants [16]. A related *Bacteroides fragilis* microvirus system shows that phiHBP1 infection drives genomic structural variation correlated with host resistance, whereas evolved phages acquire capsid and pilot-protein mutations that allow reinfection of resistant populations [17]. These examples emphasize that receptor masking, capsule switching, O-antigen variation, and phase-variable lipoprotein expression are best treated as entry-level replication-niche barriers when they alter adsorption or genome delivery. They should be classified as immune-gated only if surface remodeling is shown to be controlled by an infection-specific recognition-and-effector mechanism.

**Post-entry barriers: intracellular production constraints.** Even after a phage has successfully entered the cell, a variety of intracellular or community-derived conditions can limit productive infection. These post-entry replication-niche barriers can be broadly divided into growth-machinery limitations, chemical and ecological inhibitors, and stress-program barriers. Because these subtypes overlap, the purpose of the subclass label is not to force exclusivity, but to identify the most proximal reversible cause and the most useful engineering intervention. A defined extracellular molecule, antibiotic, metabolite, or community product is best labeled a chemical/ecological barrier, even when the downstream effect includes stress signaling. A regulated pathway such as the stringent response is best labeled a stress-program barrier when it is causally implicated in restriction and genetic, biochemical, or phage-encoded suppression restores phage production [18]. A limitation in translation capacity, nucleotide supply, energy flux, or assembly resources is best labeled a growth-machinery limitation when restriction tracks the availability of those production resources. When these causes cannot be separated experimentally, authors should use a compound label, such as post-entry replication-niche barrier involving antibiotic-induced stress and reduced translation capacity, rather than forcing a single subclass.

*Growth-machinery limitations* occur when core host functions required by both bacterium and phage are disabled, depleted, or inaccessible. If a host cell cannot sustain the biosynthetic flux required for its own growth, because nucleotides, amino acids, or energy carriers are limiting, phage production is also constrained. Dormant or persister-like cells often fall into this category: deep-dormant bacteria are frequently refractory to productive infection, and many phages can enter a hibernation-like state until the host resuscitates, although rare exceptions such as the phage Paride can replicate directly in deep-dormant *Pseudomonas aeruginosa* [19]. Nutrient deprivation experiments with T4 infecting *E. coli* under chemostat conditions confirm that host growth rate and nutrient history can strongly reshape latent period, burst size, and phage release [20]. This category should not be interpreted as "any essential process equals antiphage restriction." Cell division is a useful counterexample: although cytokinesis is essential for bacterial proliferation, several phages encode proteins that inhibit host division, often by targeting FtsZ or Z-ring formation. In T7 and T5, such inhibition has been shown to increase phage competitiveness or growth in dividing hosts, indicating that blocking a core host process can sometimes favor, rather than restrict, phage propagation [21,22]. The operational question is thus whether the specific perturbation deprives the phage of a productive factory under the tested condition.

Chemical and ecological inhibitors provide a related version of post-entry replication-niche restriction and connect this category directly to microbial ecology. In *Streptomyces*, naturally produced DNA-intercalating molecules can block phage replication, establishing that secondary metabolites can function as chemical anti-phage defenses [23]. Aminoglycoside antibiotics provide another chemical example: they can inhibit diverse phages by blocking an early step before genome replication, and supernatants from natural producers can reproduce the effect [24]. These examples often overlap with growth-machinery limitations because they interfere with DNA replication, translation, or other host functions needed by phages; however, they are best described as chemical/ecological replication-niche barriers unless a dedicated immune gate is demonstrated.

*Stress-program barriers* are conceptually different from simple growth-machinery collapse. They are regulated survival programs, not merely damaged versions of growth. The stringent response is a paradigmatic example. During nutrient limitation, the enzymes RelA and SpoT synthesize the alarmone (p)ppGpp, which reprograms transcription and metabolism to slow growth and increase stress survival [25]. The cell remains alive but becomes poorly permissive for viral production. Phage-encoded factors can specifically counteract this barrier: the T7 phage portal protein Gp8 directly binds RelA and SpoT, selectively inhibiting their alarmone synthetase activity and suppressing (p)ppGpp accumulation in vivo [18]. By releasing this stress-imposed physiological brake, the portal protein helps convert a stress-restricted bacterium back into a replication-permissive host state. Other physiological states - including biofilm-associated growth, two-component stress signaling, and kinase-regulated host pathways [26] - may eventually fall into the same conceptual space, but they should be labeled conservatively. A pathway should be called a stress-program barrier only when the stress state is native or physiologically plausible, when matched controls separate stress restriction from generic loss of viability, and when phage-encoded factors show that overcoming the pathway improves productive infection. This criterion also separates replication-niche barriers from assay-limited phenotypes: both can affect host growth, but a barrier exists in a relevant host state and is often met by specific phage countermeasures, whereas an assay-limited phenotype tracks the artificial expression format or assay context.

## 4 Assay-limited phenotypes arise when context is wrong

The third category captures cases in which apparent antiphage protection is heavily shaped by the experimental setup rather than by a native phage-host interaction. These are referred here as *assay-limited phenotypes* (or, informally, *assay mirages*), because the phenotype can look like immunity from a distance but changes, disappears, or becomes toxic once expression level, timing, host background, or infection context is corrected. It should be emphasized that "assay-limited" does not mean "unimportant" - these phenotypes often reflect real biochemical activities - but they should not be assigned an in vivo functional label without native-context evidence. The risk is greatest when candidate loci are tested only by heterologous or plasmid-borne expression, because these formats can alter expression level, timing, stoichiometry, and host physiology [3,4]. Such assays are valuable for discovery, but discovery-format evidence should not harden too quickly into a final mechanistic name.

The AcrIII-1 debate is instructive. The DUF1874-family protein SIRV1 gp29 was originally identified as AcrIII-1 and shown, under plasmid-expression and biochemical conditions, to inhibit type III CRISPR-Cas immunity by degrading the cyclic tetra-adenylate cA₄ signaling molecule produced by type III CRISPR-Cas effector complexes [27]. However, subsequent native-context work reached a different conclusion for viral infection: native SIRV1 gp29 and its close homologue SIRV2 gp37 are expressed from middle/late viral promoters and therefore too late to block CRISPR-Cas targeting at infection onset [28,29]. The exchange does not invalidate the biochemical activity or plasmid-expression phenotype, but it shows that anti-defense or anti-CRISPR labels should state the expression context, infection stage, and native viral setting in which the function has been demonstrated. From an engineering standpoint, this distinction is not a reason to discard AcrIII-1-like ring nucleases. Rather, it shows that promoter timing, expression level, and infection stage are part of the engineered function: a protein that is too late to protect its native virus may still serve as an engineered escape module if expressed early enough and at a compatible level. Thus, authors should specify whether the demonstrated function is native anti-CRISPR activity, biochemical ring-nuclease activity, plasmid-expressed anti-CRISPR activity, or an engineered early-expression counter-defense module.

Expression level creates a related problem. A focused study of multiple antiphage systems showed that increased expression can broaden the measured protection range while also causing self-inflicted toxicity or autoimmunity [30]. Thus, broader phage inhibition is not always broader native relevance; it may reflect a high-expression state that overwhelms phage escape strategies while damaging the host. This point is especially important for computational discovery pipelines. DefensePredictor and large-scale language-model-based screens have expanded discovery space dramatically [5,6], but their success makes two-step reporting more urgent: first report the screening phenotype, then assign a mechanistic label only after native context, physiology, and timing have been tested.

## 5 A practical framework for naming new systems

The decision tree in Fig. 1**a** is intended as a naming workflow rather than a rigid taxonomy. Newly identified loci or host states should first be described as candidate antiphage loci, candidate defense loci, or phage-restrictive phenotypes when the evidence consists mainly of plaque suppression, lower EOP, delayed lysis, reduced burst size, or improved host survival in a screen. The next step is not to choose an attractive noun, but to ask where infection fails - entry, intracellular immune gating, post-entry production, or assay context - and what mechanism has been demonstrated. When several mechanisms remain plausible, the label should identify the dominant experimentally supported layer; if the evidence does not separate the layers, the label should remain compound or unresolved rather than forcing a single category. This convention is especially useful for stress-coupled immune gates, surface-associated entry barriers, and assay-limited immunity or counter-defense claims, where a single restrictive endpoint can otherwise be overinterpreted. The following criteria therefore serve less as a checklist for naming a “new defense system” than as a way to decide which layer of restriction has actually been demonstrated.

For a system to be called an *immune-gated defense*, the evidence should answer four questions. First, is the system relevant in its native context? Second, have general physiological explanations been excluded, including changes in growth rate, viability, adsorption, or envelope state? Third, is there evidence that infection is blocked after entry, such as reduced phage DNA replication, transcription, virion production, burst size, or trigger-dependent host death? Finally, is the immune gate mechanistically defined by catalytic mutants, trigger mutants, phage escapers, direct binding, or a clear self/non-self discrimination rule?

For *replication-niche barriers*, the key question is where permissivity fails and whether that failure reflects a native or physiologically plausible host or community state. Entry-level barriers require direct evidence for an adsorption, receptor-access, or genome-delivery block, ideally with restoration by receptor engineering, surface remodeling, or a phage factor. Post-entry barriers require evidence that the intracellular environment is non-permissive despite successful genome delivery, and that restriction is relieved when the limiting factor, inhibitory chemistry, metabolic state, or stress program is removed or counteracted. Native context, matched-physiology controls, and phage-encoded niche-remodeling factors separate a genuine replication-niche barrier from an assay-limited phenotype.

In reporting, each study should specify three fields separately: the phenotype (EOP reduction, delayed lysis, reduced burst size, improved host survival), the infection stage affected (adsorption, genome entry, genome replication, transcription, translation, assembly, release, or cell survival), and the mechanism (immune gating, entry-level niche barrier, post-entry growth-machinery limitation, stress-program barrier, assay-limited phenotype, or unresolved candidate). Authors should likewise specify what a counter-defense or pro-infection protein is countering: an immune gate, a receptor barrier, a chemical inhibitor, a limiting host machine, or a global stress program. These are different evolutionary stories and demand different experimental and engineering strategies.

## 6 Beyond strict boundaries: interplay, trade-offs, and why precise labels matter

The three categories proposed here are intended to clarify mechanism, but real infections rarely fall into a single box. The same bacterial cell can carry immune-gated defenses while also occupying a physiological state that limits phage adsorption, genome delivery, or intracellular production. This layered structure is the reason precise labels matter: surface states act upstream of immunity by determining whether phages ever reach the cytoplasm, whereas post-entry physiological states act alongside or downstream of immunity by determining whether virion production is possible.

Layering is also temporal and life-cycle dependent. In *Listeria seeligeri*, a native type VI-A CRISPR-Cas13 system restricts lytic replication of temperate phages while tolerating prophage acquisition and altering prophage induction [31]. Chronic viruses pose a different timing problem: the archaeal chronic virus SMV1a can evade *Saccharolobus islandicus* Argonaute immunity through early progeny release rather than direct immune inhibition [32]. These examples show that immunity, escape, and restriction depend not only on mechanism, but also on life-cycle stage, expression timing, and the endpoint being measured.

The layers can also regulate one another. Replication-niche barriers are not merely parallel to immune defenses; stress, nutrient limitation, chemical exposure, envelope state, and metabolic state can change the expression, abundance, or activation thresholds of immune-gated systems. This possibility is illustrated by kinase- and two-component-system examples. In *Serratia* sp. ATCC 39,006, activation of the Rcs envelope-stress pathway promotes surface changes that block phage adsorption while suppressing CRISPR-Cas adaptive immunity. Conversely, in *Marinomonas mediterranea*, the BarA/UvrY-like PpoS/PpoR system transcriptionally upregulates dual CRISPR-Cas systems and primes phage resistance. These examples show that host physiological states can couple infection permissivity with immune readiness, changing both whether phages can enter or replicate and how strongly immune-gated defenses are expressed or triggered [26].

A concrete example of layered restriction comes from recent work on T-even phage infection of wild *E. coli* [15]. In that study, O-antigen barriers could obscure intracellular defense phenotypes; once these surface barriers were removed or experimentally bypassed, GmrSD-family type IV restriction enzymes became visible as a second layer of restriction. T4 counters this intracellular layer with capsid-packaged internal proteins, including Ip2 and Ip3, that inhibit distinct GmrSD variants. This experimental “peeling back” of layers demonstrates that entry barriers and immune-gated restriction can stack in series, and that an unmeasured surface barrier can hide the intracellular immune repertoire in bulk infection assays.

This complexity strengthens, rather than weakens, the three-way classification. Many replication-niche barriers function as ecological “defenses” in the broad sense because they reduce the probability of productive infection in natural populations. However, the mechanistic label “immune-gated defense” should be reserved for systems in which restriction is licensed by a recognition event. This distinction has practical consequences. A surface polysaccharide that blocks adsorption is overcome by receptor-binding protein changes, depolymerases, or surface remodeling, not by an anti-CRISPR (Fig. 1**c**). A stress-restricted or nutrient-limited host requires niche remodeling, not only immune evasion. Conversely, a true immune gate calls for trigger mapping, trigger avoidance, or anti-defense proteins. Clear labels therefore improve annotation, sharpen evolutionary interpretation, and make phage engineering more rational [33].

The familiar parallel between bacterial defense and eukaryotic innate immunity is strongest when bacterial systems genuinely exhibit immune gating. A complementary parallel is now equally useful: as in bacterial pathogenesis, successful phage infection requires both avoidance of host defenses and construction of a permissive replication niche. This matters because bacterial life is often stressful, slow, and heterogeneous. When phage restriction is not immunity, it may still reveal important biology and, from an engineering standpoint, may point to host processes that are more tractable targets for phage-based interventions than the immune systems themselves. The most useful engineering question is therefore not simply whether a bacterium is resistant, but which layer of the infection pathway prevents productive replication.

## Declaration of generative AI and AI-assisted technologies in the manuscript preparation process

During the preparation of this work the author used ChatGPT (OpenAI, GPT-4o) to improve the English language and organization of the manuscript. After using this tool, the author reviewed and edited the content as needed and takes full responsibility for the content of the published article.

## CRediT authorship contribution statement

**Yong Everett Zhang:** Writing – review & editing, Writing – original draft, Visualization, Investigation, Funding acquisition, Conceptualization.

## Declaration of Competing Interest

The authors declare that they have no known competing financial interests or personal relationships that could have appeared to influence the work reported in this paper.

The author YONG Everett ZHANG is an Editorial Board Member for Engineering Microbiology and was not involved in the editorial review or the decision to publish this article.

## Appendix Supplementary materials

Image, application 1
